# Incident heart failure, arrhythmias and cardiovascular outcomes with sodium‐glucose cotransporter 2 (SGLT2) inhibitor use in patients with diabetes: Insights from a global federated electronic medical record database

**DOI:** 10.1111/dom.14854

**Published:** 2022-09-27

**Authors:** Ameenathul Mazaya Fawzy, José Miguel Rivera‐Caravaca, Paula Underhill, Laurent Fauchier, Gregory Y. H. Lip

**Affiliations:** ^1^ Liverpool Centre for Cardiovascular Science University of Liverpool and Liverpool Heart & Chest Hospital Liverpool UK; ^2^ Department of Cardiology, Hospital Clínico Universitario Virgen de la Arrixaca University of Murcia, Instituto Murciano de Investigación Biosanitaria (IMIB‐Arrixaca), CIBERCV Murcia Spain; ^3^ TriNetX LLC London UK; ^4^ Service de Cardiologie Centre Hospitalier Universitaire Trousseau Tours France; ^5^ Department of Clinical Medicine Aalborg University Aalborg Denmark

**Keywords:** cardiovascular outcomes, diabetic, incident heart failure, prognosis, SGLT2 inhibitors

## Abstract

**Aim:**

To investigate the impact of sodium‐glucose cotransporter 2 (SGLT2) inhibitors on the risk of incident heart failure and adverse cardiovascular outcomes.

**Methods:**

All patients with diabetes who were registered between January 2018 and December 2019 were identified from a federated electronic medical record database (TriNetX) and followed up for 2 years. A 1:1 propensity‐score matching (PSM) analysis was performed to balance the SGLT2 inhibitor and non‐SGLT2 inhibitor cohorts. The primary outcome was incident heart failure. Secondary outcomes included all‐cause mortality, cardiac arrest, ventricular tachycardia/ventricular fibrillation (VT/VF), incident atrial fibrillation (AF), ischaemic stroke/transient ischaemic attack (TIA), composite of arterial and venous thrombotic events, and composite of incident VT/VF and cardiac arrest.

**Results:**

A total of 131 189 and 2 692 985 patients were treated with and without SGLT2 inhibitors, respectively. After PSM, 131 188 patients remained in each group. The risk of incident heart failure was significantly lower in the SGLT2 inhibitor cohort compared to the non‐SGLT2 inhibitor cohort (hazard ratio [HR] 0.70, 95% confidence interval [CI] 0.68‐0.73). SGLT2 inhibitor use was also associated with a significantly lower risk of all‐cause mortality (HR 0.61, 95% CI 0.58‐0.64), cardiac arrest (HR 0.70, 95% CI 0.63‐0.78), incident AF (HR 0.81, 95% CI 0.76‐0.84), ischaemic stroke/TIA (HR 0.90, 95% CI 0.88‐0.93), composite of arterial and venous thrombotic events (HR 0.90, 95% CI 0.88‐0.92), and composite of incident VT/VF and cardiac arrest (HR 0.76, 95% CI 0.71‐0.81). There were no significant differences for VT/VF (HR 0.94, 95% CI 0.88‐1.00).

**Conclusion:**

Use of SGLT2 inhibitors was associated with a significant reduction in the risk of incident heart failure and adverse cardiovascular outcomes but not ventricular arrhythmias.

## 1 INTRODUCTION

Sodium‐glucose cotransporter 2 (SGLT2) inhibitors gained universal prominence across the cardiac community and recently became incorporated into guidelines as a treatment of choice for heart failure with reduced ejection fraction (HFrEF), after their use as antidiabetic drugs for many years.^1^, ^2^ Indeed, many studies in patients with diabetes treated with SGLT2 inhibitors, have consistently demonstrated lower rates of adverse cardiovascular outcomes with these drugs.^3^, ^4^, ^5^

In the DAPA‐HF study, which randomized HFrEF patients to either a dapagliflozin or a placebo arm, a statistically significant reduction in cardiovascular mortality and hospitalization for heart failure exacerbations was observed in patients treated with the former, regardless of the presence or absence of diabetes.^1^ Subsequent studies have reflected similar findings for heart failure outcomes, with conflicting results for some of the other outcomes.^6^, ^7^ Notably, these observations were made even in those without any established cardiovascular disease, suggesting that these drugs may have a role in prevention, or at least slowing progression, of certain conditions such as heart failure. Furthermore, in all the randomized controlled trials (RCTs) the beneficial effects were observed within a matter of weeks, regardless of glycaemic control, suggesting that SGLT2 inhibitors exert their effects through glucose‐independent mechanisms.

Although the beneficial effects of SGLT2 inhibitors on heart failure outcomes have been demonstrated consistently, their impact on some of the other cardiovascular outcomes are less apparent, owing to mixed evidence from RCTs and limited observational studies. Thus, this study aimed to: (a) evaluate the association between SGLT2 inhibitors and incident heart failure, as well as other non‐heart‐failure‐related adverse cardiovascular events such as mortality, arrhythmias and vascular thrombosis, using a large global federated database and (b) determine whether these results from a real‐world population were consistent with those of RCTs such as EMPA‐REG OUTCOME and DECLARE‐TIMI 58, which were conducted in selective study populations under stringent conditions.

## 2 METHODS

### 2.1 Study design and population

This retrospective, observational cohort study was conducted using the TriNetX research network, a global federated administrative database with real‐time updates of electronic medical records (EMRs). It holds data on approximately 85 million patients from >60 healthcare organizations across seven countries, with the majority of centres based in the United States. Other participating countries are Germany, the United Kingdom, Italy, Singapore and Israel. A detailed description of the database is provided in the paper by Topaloglu et al^8^ and can be found at the website: https://trinetx.com/company-overview/.

Briefly, the TriNetX research network database encompasses anonymized EMRs of patients registered with the network and has information on patient demographics, clinical details including diagnoses, medications and investigations as well as any procedures, from settings such as general practice surgeries, and community and secondary hospitals, providing detailed real‐world data.

**FIGURE 1:**
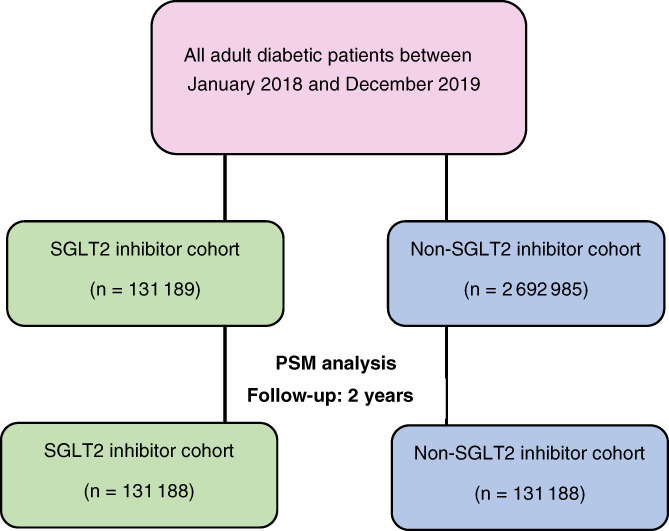
Study selection process. PSM, propensity‐score matching; SGLT2, sodium‐glucose cotransporter 2 inhibitor

All registered adult patients with type 2 diabetes between January 1, 2018 and December 31, 2019 were identified from the TriNetX database using the International Classification of Diseases, Tenth Revision (ICD‐10) code, E11. Patients were categorized into two groups depending on treatment with or without SGLT2 inhibitors (Figure 1). Patients not receiving SGLT2 inhibitors (either empagliflozin, dapagliflozin or canagliflozin) at inclusion who were subsequently prescribed it were censored when the drug was initiated. The searches were run on December 27, 2021. At the time of the search, there were 57 participating healthcare organizations within the TriNetX research network.

As TriNetX only provides access to de‐identified data, research studies conducted using TriNetX do not require ethical approval. Nonetheless, extensive data quality assessments are performed to ensure adherence of the platform to the institutional review board requirements and conformance, completeness and plausibility of the available data.^8^, ^9^ Study proceedings were carried out according to the Declaration of Helsinki.

### 2.2 Follow‐up and outcomes

All patients were followed up for 2 years. During this period, follow‐up data were collected for the primary outcome of incident heart failure (ICD‐10‐CM code: I50) and secondary outcomes, which included all‐cause mortality, incident atrial fibrillation (AF; ICD‐10‐CM code: I48), incident ventricular tachycardia (VT)/ventricular fibrillation (VF; ICD‐10‐CM codes: I47.2 or I49.0), cardiac arrest (ICD‐10‐CM code: I46), ischaemic stroke/transient ischaemic attack (TIA; ICD‐10‐CM codes: G45 or I63), a composite of incident VT/VF/cardiac arrest using the corresponding ICD‐10 codes, and a composite of arterial and venous thrombotic events (ICD‐10‐CM codes: G45 [transient cerebral ischaemic attacks and related syndromes], I63 [cerebral infarction], I67.82 [cerebral ischaemia], I74 [arterial embolism and thrombosis], I26 [pulmonary embolism], I81 [portal vein thrombosis], I82 [other venous embolism and thrombosis], I21 [acute myocardial infarction], or I22 [subsequent ST elevation and non‐ST elevation myocardial infarction]).

### 2.3 Statistical analysis

Baseline characteristics were represented using continuous variables, expressed as mean (± standard deviation [SD]), or categorical variables, expressed as counts and percentages. Differences between groups were assessed using Student′s *t*‐test for continuous variables and the chi‐squared test for categorical variables.

To account for the large differences in the number of patients between the two comparator groups as well as the baseline characteristics and potential confounding effects, data analysis was performed using propensity‐score matching (PSM) using “greedy nearest‐neighbour matching” (with a caliper of 0.1 of pooled SDs and difference between propensity scores ≤0.1). PSM was calculated using the covariates shown in Table 1. Standardized mean differences (SMDs) were used to exhibit the distribution of demographic data and clinical characteristics between the two groups and calculated as the difference in the means or proportions of a particular variable divided by the pooled estimate of SD for that variable. Any baseline characteristic with an SMD of <0.1 between cohorts was considered well matched.

**TABLE 1:** Baseline characteristics of the two groups before and after matching

	Before matching				After matching			
	Non‐SGLT2 inhibitor (n = 2 692 985)	SGLT2 inhibitor (n = 131 189)	*P* value	SMD	Non‐SGLT2 inhibitor (n = 131 188)	SGLT2 inhibitor (n = 131 188)	*P* value	SMD
Age, years, mean ± SD	60.60 ± 15.81	58.38 ± 11.38	<0.001	0.161	58.38 ± 11.38	58.79 ± 13.72	<0.001	0.032
Female gender, n (%)	1 386 014 (51.47)	57 529 (43.85)	<0.001	0.152	57 529 (43.85)	58 013 (44.22)	0.056	0.007
Ethnicity, n (%)								
Caucasian	1 720 648 (63.89)	92 109 (70.21)	<0.001	0.134	92 108 (70.21)	91 894 (70.05)	0.361	0.003
Black	503 747 (18.71)	19 315 (14.72)	<0.001	0.106	19 315 (14.72)	19 698 (15.02)	0.035	0.008
Asian	76 396 (2.84)	3564 (2.72)	0.010	0.007	3564 (2.72)	3585 (2.73)	0.801	<0.001
Not specified	373 539 (13.87)	15 413 (11.75)	<0.001	0.063	15 413 (11.75)	15 100 (11.51)	0.056	0.007
Comorbidities and laboratory results								
Glucose, mmol, mean ± SD	8.48 ± 4	9.79 ± 4.30)	<0.001	0.375	9.79 ± 4.30	9.03 ± 4.4	<0.001	0.015
HbA1c, %, mean ± SD	7.40 ± 1.99	8.41 ± 1.84	<0.001	0.524	8.41 ± 1.84	7.72 ± 1.97	<0.001	0.021
Hypertension, n (%)	1 929 831 (71.66)	103 630 (78.99)	<0.001	0.170	103 629 (78.99)	104 075 (79.33)	0.032	0.008
Dyslipidaemia, n (%)	1 523 541 (56.57)	101 010 (77.00)	<0.001	0.444	101 009 (77.00)	102 006 (77.76)	<0.001	0.018
Total cholesterol, mmol/L, mean ± SD	4.39 ± 1.23	4.31 ± 1.25	<0.001	0.491	4.31 ± 1.25	4.37 ± 1.25	<0.001	0.018
Triglycerides, mmol/L, mean ± SD	1.85 ± 1.79	2.24 ± 2.39	<0.001	0.513	2.24 ± 2.39	2.09 ± 2.28	<0.001	0.015
LDL cholesterol, mmol/L, mean ± SD	2.41 ± 0.97	2.28 ± 0.96	<0.001	0.474	2.28 ± 0.96	2.37 ± 0.97	<0.001	0.019
HDL cholesterol, mmol/L, mean ± SD	1.20 ± 0.41	1.10 ± 0.35	<0.001	0.482	1.10 ± 0.35	1.14 ± 0.37	<0.001	0.010
Lung conditions, n (%)	1 242 947 (46.16)	65 834 (50.18)	<0.001	0.080	65 833 (50.18)	66 063 (50.36)	0.369	0.003
Body mass index, kg/m^2^, mean ± SD	31.44 ± 7.33	33.84 ± 6.69	<0.001	0.027	33.84 ± 6.69	33.01 ± 7.03	<0.001	0.020
Obesity, n (%)	792 900 (29.44)	57 897 (44.13)	<0.001	0.308	57 896 (44.13)	57 499 (43.83)	0.118	0.006
Neoplasms, n (%)	756 430 (28.09)	40 330 (30.74)	<0.001	0.058	40 329 (30.74)	40 283 (30.71)	0.845	<0.001
IHD, n (%)	634 392 (23.56)	33 129 (25.25)	<0.001	0.039	33 128 (25.25)	33 204 (25.31)	0.732	0.001
Kidney disease, n (%)	578 179 (21.47)	17 901 (13.65)	<0.001	0.206	17 901 (13.65)	18 293 (13.94)	0.026	0.008
Liver disease, n (%)	284 338 (10.56)	18 129 (13.82)	<0.001	0.099	18 128 (13.82)	17 700 (13.49)	0.014	0.009
Heart failure, n (%)	337 879 (12.55)	12 764 (9.73)	<0.001	0.089	12 764 (9.73)	12 988 (9.90)	0.141	0.005
Cerebrovascular disease, n (%)	333 649 (12.39)	13 349 (10.18)	<0.001	0.070	13 349 (10.18)	13 487 (10.28)	0.373	0.003
Atrial fibrillation, n (%)	273 244 (10.15)	10 002 (7.62)	<0.001	0.088	10 002 (7.62)	10 105 (7.70)	0.449	0.002
Peripheral vascular disease, n (%)	175 731 (6.53)	8604 (6.56)	0.636	0.001	8604 (6.56)	8543 (6.51)	0.629	0.001
Medications, n (%)								
Metformin	936 361 (34.77)	104 638 (79.76)	<0.001	1.021	104 637 (79.76)	107 045 (81.60)	<0.001	0.046
Statins	1 170 177 (43.45)	101 301 (77.22)	<0.001	0.735	101 300 (77.22)	103 026 (78.53)	<0.001	0.031
ACE inhibitors	781 941 (29.04)	64 786 (49.38)	<0.001	0.426	64 786 (49.38)	66 188 (50.45)	<0.001	0.021
Insulin	874 279 (32.47)	63 502 (48.41)	<0.001	0.329	63 501 (48.41)	62 993 (48.02)	0.047	0.007
Anti‐platelets	846 937 (31.45)	55 566 (42.36)	<0.001	0.227	55 565 (42.36)	56 658 (43.19)	<0.001	0.016
Diuretics	860 988 (31.97)	55 694 (42.45)	<0.001	0.218	55 693 (42.45)	56 585 (43.13)	<0.001	0.013
Beta‐blockers	880 313 (32.69)	54 547 (41.58)	<0.001	0.184	54 547 (41.58)	55 478 (42.29)	<0.001	0.014
AADs	790 173 (29.34)	42 463 (32.37)	<0.001	0.065	42 462 (32.37)	42 499 (32.40)	0.877	0.001
ARBs	434 357 (16.13)	39 304 (29.96)	<0.001	0.332	39 303 (29.96)	39 098 (29.80)	0.381	0.003
CCBs	616 609 (22.90)	37 203 (28.36)	<0.001	0.125	37 203 (28.36)	37 900 (28.89)	0.002	0.011
Anticoagulation	715 769 (26.58)	34 000 (25.92)	<0.001	0.015	34 000 (25.92)	34 626 (26.39)	0.005	0.010
Sitagliptin	154 065 (5.72)	33 008 (25.16)	<0.001	0.558	33 007 (25.16)	30 222 (23.04)	<0.001	0.049
Glipizide	224 579 (8.34)	27 899 (21.27)	<0.001	0.370	27 898 (21.27)	27 197 (20.73)	<0.001	0.013

Abbreviations: AAD, anti‐arrhythmic drug; ACE, angiotensin‐converting enzyme; ARB, angiotensin receptor blocker; CCB, calcium channel blockers; IHD, ischaemic heart disease; SD, standard deviation; SGLT2, sodium glucose cotransporter 2; SMD, standardized mean difference.

Cox regression models were used to evaluate the association between treatment with or without SGLT2 inhibitors and the outcomes of interest. Where incident outcomes were evaluated, patients with a pre‐existing history of the outcome of interest were excluded from that particular analysis. For example, patients with prevalent heart failure were excluded when examining incident heart failure. Results were expressed as hazard ratios (HRs) with 95% confidence intervals (CIs). No imputations were made for missing data.

All tests were two‐tailed and *P* values of ≤0.05 were taken to indicate statistical significance. Statistical analysis was performed on the TriNetX Analytics platform which provides an interface with real‐time browser‐based analytics features. Results are presented according to the Strengthening the Reporting of Observational Studies in Epidemiology (STROBE) reporting guidelines (Table S1).

## 3 RESULTS

A total of 2 824 174 patients with diabetes were identified during the specified study period. Of these, 2 692 985  patients were not on SGLT2 inhibitors (mean age 60.60 ± 15.81 years) and only 131 189 were treated with SGLT2 inhibitors (mean age 58.38 ± 11.38 years). Over three‐quarters of patients in both the groups had hypertension and lipid disorders. The SGLT2 inhibitor group had a higher proportions of patients with ischaemic heart disease, and liver and respiratory disorders but a lower proportion of prevalent heart failure and AF (Table 1). Those in the SGLT2 inhibitor group were also more likely to be on other antidiabetic drugs including metformin, sulphonylureas, such as glipizide, dipeptidyl peptidase‐4 (DPP‐4) inhibitors, such as sitagliptin, and insulin, as well as cardiac drugs, indicative of a more complex cohort of patients.

Due to the significant discrepancies in cohort sizes and baseline characteristics as described above, PSM was carried out on a 1:1 basis, with 131 188 patients in both groups. After PSM, the mean ages of the population in the non‐SGLT2 inhibitor and SGLT2 inhibitor groups were 58.79 ± 13.72 and 58.38 ± 11.38 years, respectively. The non‐SGLT2 inhibitor group had a higher proportion of patients with dyslipidaemia and higher mean total, HDL and LDL cholesterol levels, whereas triglyceride levels were higher in the SGLT2 inhibitor cohort, which also had higher glycated haemoglobin (HbA1c) levels. The mean body mass index was also higher in this group. However, the SMD for all of the variables assessed was <0.1, hence, the differences were considered marginal. [Correction added on 04 November 2022, after first online publication: The non‐SGLT2 inhibitor and SGLT2 inhibitor texts were previously swapped and have now been corrected.]

### 3.1 Incident heart failure

Of the 262 378 patients analysed (*n* = 131 188 in each group), 10 775 patients from the non‐SGLT2 inhibitor group and 11 567 patients from the SGLT2 inhibitor group were excluded due to a known history of heart failure prior to the period of observation. As such, 120 413 patients from the non‐SGLT2 inhibitor group and 119 621 from the SGLT2 inhibitor group were included in the analysis for the primary outcome (Table 2).

**TABLE 2:** Outcome events in the patients included from the propensity‐score‐matched analysis

Outcome	SGLT2 inhibitor group			Non‐SGLT2 inhibitor group		
	Included, n (%)	Excluded, n (%)	Events, n (%)	Included, n (%)	Excluded, n (%)	Events, n (%)
Incident heart failure	119 621 (91.18)	11 567 (8.82)	5717 (4.78)	120 413 (91.79)	10 775 (8.21)	7887 (6.55)
Incident AF	122 342 (93.26)	8846 (6.74)	4451 (3.64)	122 807 (93.61)	8381 (6.39)	5388 (4.39)
VT/VF	131 188 (100.0)	—	2114 (1.61)	131 188 (100.0)	‐	2182 (1.66)
Cardiac arrest	130 805 (99.70)	383 (0.29)	656 (0.50)	130 807 (99.71)	381 (0.29)	902 (0.69)
Composite of VT/VF/cardiac arrest	128 832 (98.20)	2356 (1.80)	1595 (1.24)	129 041 (98.36)	2147 (1.64)	2048 (1.59)
All‐cause mortality	131 188 (100.0)	—	2744 (2.09)	131 188 (100.0)	—	4364 (3.33)
Ischaemic stroke	131 188 (100.0)	—	9300 (7.09)	131 188 (100.0)	—	10 060 (7.67)
Composite of venous and arterial thrombosis	131 188 (100.0)	—	17 987 (13.71)	131 188 (100.0)	—	19 394 (14.78)

Abbreviations: AF, atrial fibrillation; SGLT2, sodium glucose cotransporter 2; VF, ventricular fibrillation; VT, ventricular tachycardia.

**FIGURE 2:**
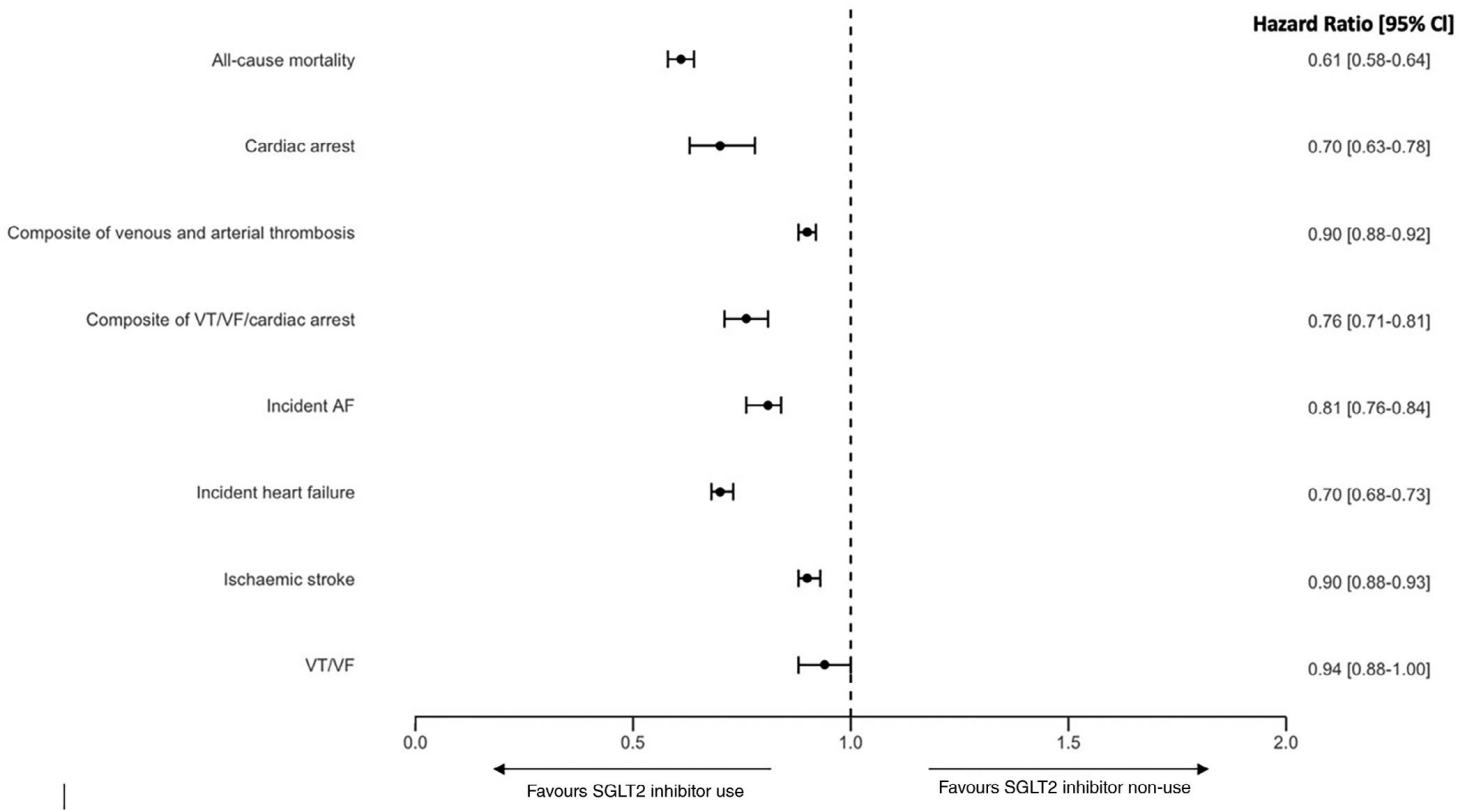
Forest plot demonstrating risk of outcomes associated with sodium‐glucose cotransporter 2 (SGLT2) inhibitor use. AF, atrial fibrillation; VF, ventricular fibrillation; VT, ventricular tachycardia

During the 2‐year follow‐up period, 7887 (6.55%) and 5717 (4.78%) diagnoses of incident heart failure were made in the non‐SGLT2 inhibitor and SGLT2 inhibitor groups, respectively. SGLT2 inhibitor use was associated with a significantly lower risk of incident heart failure (HR 0.70 [95% CI 0.68‐0.73]; log‐rank *P* value <0.0001 [Figure 2]).

### 3.2 All‐cause mortality

A total of 4364 and 2744 deaths (Table 2) were observed in the non‐SGLT2 inhibitor and SGLT2 inhibitor groups, respectively. The risk of all‐cause mortality was significantly lower with SGLT2 inhibitor use, with an HR of 0.61 (95% CI 0.58‐0.64; log‐rank *P*‐value 0.008 [Table 3; Figure 2]).

**TABLE 3:** Risk of outcome events between the two groups

Outcome	Total	SGLT2 inhibitor use, n (%)	SGLT2 inhibitor non‐use, n (%)	HR (95% CI)	*P* value
Incident heart failure	13 604	5717 (4.78)	7887 (6.55)	0.70 (0.68‐0.73)	<0.0001
Incident AF	9839	4451 (3.64)	5388 (4.39)	0.81 (0.76‐0.84)	<0.0001
VT/VF	4296	2114 (1.61)	2182 (1.66)	0.94 (0.88‐1.00)	0.0551
Cardiac arrest	1558	656 (0.50)	902 (0.69)	0.70 (0.63‐0.78)	0.001
Composite of VT/VF/cardiac arrest	3643	1595 (1.24)	2048 (1.59)	0.76 (0.71‐0.81),	0.0059
All‐cause mortality	7108	2744 (2.09)	4364	0.61 (0.58‐0.64)	0.0008
Ischaemic stroke	19 360	9300 (7.09)	10 060 (7.67)	0.90 (0.88‐0.93)	<0.0001
Composite of venous and arterial thrombosis	37 281	17 987 (13.71)	19 394 (14.78)	0.90 (0.88‐0.92)	<0.0001

Abbreviations: AF, atrial fibrillation; CI, confidence interval; HR, hazard ratio; SGLT2, sodium‐glucose cotransporter 2; VF, ventricular fibrillation; VT, ventricular tachycardia. [Correction added on 04 November 2022, after first online publication: The P value of VT/VF has been corrected.]

### 3.3 Arrhythmias

#### 3.3.1 Incident AF

After excluding 8381 patients from the non‐SGLT2 inhibitor group and 8846 patients from the SGLT2 inhibitor group due to history of AF prior to the observation period (Table 2), SGLT2 inhibitor use was associated with a 19% lower risk of incident AF (HR 0.81 [95% CI 0.76‐0.84]; log rank *P* value <0.0001) and longer AF‐free survival.

#### 3.3.2 Ventricular tachycardia/ventricular fibrillation

During the 2 years of follow‐up, 2182 patients from the non‐SGLT2 inhibitor group and 2114 from the SGLT2 inhibitor group had documented episodes of VT or VF. Although SGLT2 inhibitor use was associated with a lower risk of these events, this result was not statistically significant (HR 0.94 [95% CI 0.88‐1.00]; log‐rank *P* value = 0.628 [Table 3]), indicating that they had no effect on ventricular arrhythmias.

#### 3.3.3 Cardiac arrest

A total of 381 patients from the non‐SGLT2 inhibitor cohort and 383 patients from the SGLT2 inhibitor cohort had a history of cardiac arrest prior to the observation period. Thus, 130 807 patients from the non‐SGLT2 inhibitor group and 130 805 from the SGLT2 inhibitor group were included in the analysis for this outcome event (Table 2). SGLT2 inhibitor use was associated with a significantly lower number of cardiac arrests (656 vs. 902) compared to non‐use (HR 0.70 [95% CI 0.63‐0.78]; log‐rank *P* value 0.001 [Table 3]).

#### 3.3.4 Composite of incident VT, VF and cardiac arrest

A total of 2147 patients in the non‐SGLT2 inhibitor cohort and 2356 in the SGLT2 inhibitor group who had a history of the outcome were excluded. SGLT2 inhibitor use was associated with a significantly lower risk of the composite outcome of ventricular arrhythmias and cardiac arrests, with an HR of 0.76 (95% CI 0.71‐0.81; log‐rank *P* value 0.0059 [Table 3]). This was predominantly driven by the significant reduction in cardiac arrests rather than ventricular arrhythmias per se.

### 3.4 Ischaemic stroke/TIA

Compared to patients in the non‐SGLT2 inhibitor group, those in the SGLT2 inhibitor group had a significantly lower incidence of ischaemic strokes/TIA (7.67% vs. 7.01%), corresponding with a 10% lower risk (HR 0.90 [95% CI 0.88‐0.93]; log‐rank *P* value <0.0001 [Table 3]).

### 3.5 Composite of venous and arterial thrombotic events

A total of 19 394 venous and arterial thrombotic events were observed in the non‐SGLT2 inhibitor group, and 17 987 in the SGLT2 inhibitor group. Overall, the risk for the composite of venous and arterial thrombotic events was significantly lower in the SGLT2 inhibitor cohort (HR 0.90 [95% CI 0.88‐0.92]; log‐rank *P* value <0.0001).

## 4 DISCUSSION

The principal findings from our study are that SGLT2 inhibitor use was associated with the following: (a) a 30% lower risk of incident heart failure; (b) a significantly lower risk of AF and cardiac arrests, but not ventricular arrhythmias; and (c) a significant reduction in the risk of other secondary outcome events, such as all‐cause mortality, ischaemic strokes/TIA and the composite of arterial and venous thrombotic events.

These findings support the existing notion that the benefits of SGLT2 inhibitors extend beyond glycaemic control and heart failure outcomes and that they exert their effects through other mechanisms. Furthermore, these benefits were observed within a short duration of time, as seen with previous studies.

Despite the many theories that have been postulated, the exact mechanisms by which SGLT2 inhibitors bring about their effects are not fully understood. The most widely accepted concept for the reduction in heart failure hospitalizations is attributed to the reduction in the effective circulating volume owing to increased diuresis related to glycosuria as well as the natriuresis, with SGLT2 inhibitor use; however, this explanation is believed to be incomplete as these effects are transient with a return towards baseline within a few days due to re‐establishment of the sodium‐water balance and studies suggesting no significant reduction in urinary excretion.^10^, ^11^ In contrast, other studies have demonstrated reductions in plasma volume by up to 8%, with these changes sustained even at 6 months.^12^, ^13^ In secondary analyses of the EMPA‐REG‐OUTCOME trial and the CANVAS program, changes in markers associated with plasma volume such as haemoglobin, haematocrit and amino‐terminal pro–B‐type natriuretic peptide (NT‐pro BNP) were the most significant mediators of cardiovascular mortality and heart failure hospitalizations.^5^, ^14^

Additionally, SGLT2 inhibitors have been shown to influence biomarkers such as leptin, ketone bodies, uric acid advanced glycation end‐products as well as ion transporters that have adverse effects on the myocardium and the vasculature through oxidative and inflammatory processes, resulting in deleterious consequences such as myocardial fibrosis, ventricular remodelling and arterial stiffness, increasing the risk of coronary artery disease, myocardial infarction, arrhythmogenesis and heart failure.^11^ Thus, it is likely that the influence of SGLT2 inhibitors on these mechanisms may play a role in the prevention of or delay in the development of heart failure and other adverse cardiovascular events.

In this PSM analysis, SGLT2 inhibitor use was associated with a 30% risk reduction of incident heart failure. This is congruent with the early RCTs, where a potential preventative role was insinuated due to the observation of lower hospitalization rates for heart failure despite the absence of pre‐existing heart failure in the majority (>85%) of participants.^3^, ^4^, ^5^ In a subgroup analysis of the DECLARE‐TIMI 58 trial, the risk of the composite outcome of cardiovascular death and/or hospitalization for heart failure was significantly lower in the dapagliflozin group compared to placebo in the subgroups with and without prevalent heart failure.^3^ Nonetheless, none of the RCTs thus far have examined incident heart failure as a specific endpoint.

Observational studies have, however, explored the potential preventative effects of SGLT2 inhibitors. In the CVD‐REAL study, which was a multi‐national study based on electronic records and claims databases, SGLT2 inhibitor use was associated with a significant reduction in the risk of mortality and heart failure, regardless of pre‐existing cardiovascular disease.^15^ Similarly, in the study by Zhou et al in a Chinese population, those treated with SGLT2 inhibitors had a 27% lower risk of developing new‐onset heart failure, as well as reductions in the risk of all‐cause mortality and myocardial infarction.^16^

Birkeland et al demonstrated a similar 29% reduction in the risk of incident heart failure in a population with type 2 diabetes without prevalent cardiovascular or renal disease^17^ but showed no difference in the risk of myocardial infarction. Further, the risk of ischaemic stroke was significantly lower in the SGLT2 inhibitor group, as in our study, which demonstrated a 10% lower risk. These findings differ from those of the EMPRISE study^18^ and a contemporary meta‐analysis which indicated that SGLT2 inhibitors have negligible effects on the risk of stroke, regardless of subtype. That said, a risk reduction of 50% was observed with SGLT2 inhibitors when haemorrhagic strokes were factored in, suggesting a potential protective effect.^19^ In the CVD‐REAL‐2 study, which focused on Asia Pacific, Middle Eastern and North American populations, significant reductions in all the aforementioned outcomes were noted in patients with diabetes treated with SGLT2 inhibitors compared with other glucose‐lowering therapies.^20^

In addition to the above, SGLT2 inhibitors have also been associated with AF risk reduction. This was evident from a post hoc analysis of the DECLARE‐TIMI 58 trial and experimentally supported by a canine study in which canagliflozin suppressed atrial remodelling.^21^, ^22^ A meta‐analysis of existing RCTs suggests that SGLT2 inhibitors may indeed be associated with a lower risk of incident AF (odds ratio 0.82 [95% CI 0.72‐0.93]; *P* = 0.002), consistent with our study.^23^

While the potential antiarrhythmic mechanisms of SGLT2 inhibitors^11^, ^24^, ^25^ should theoretically result in a reduction of both supraventricular and ventricular arrhythmias, the bulk of the evidence from RCTs suggests that SGLT2 inhibitors do not affect ventricular arrhythmias or sudden cardiac death.^26^, ^27^ In one meta‐analysis, however, a significantly lower risk of VT was observed (relative risk 0.73 [95% CI 0.53‐0.99]).^28^ Our findings were consistent with the former, showing no significant impact on ventricular arrhythmias, although a marked risk reduction in cardiac arrests was seen. There was also a significant reduction in the composite outcome of VT/VF and cardiac arrests, primarily driven by the reduced rates of cardiac arrests.

Given the speculations that SGLT2 inhibitors may increase the risk of thromboembolism due to the increase in haematocrit and blood viscosity, we also looked at the composite of arterial and venous thrombosis rates associated with SGLT2 inhibitor use. Not only did the findings show that there was no increased risk but they suggested a potential protective effect against arterial and venous thrombotic events. In the meta‐analysis of RCTs there was no association between SGLT2 inhibitor use and the risk of venous thromboembolism including deep vein thromboses and pulmonary emboli (relative risk 0.98 [95% CI, 0.75‐1.28]), but results from observational studies have been mixed.^29^, ^30^, ^31^

Studies focusing on arterial thrombosis with SGLT2 inhibitors have been limited, but in another meta‐analysis by Lin et al.^32^ that assessed lower limb complications, the risk of peripheral arterial disease events, which comprised arterial thrombosis, was significantly elevated with SGLT2 inhibitor use. Subgroup analysis according to drug type revealed that this was only observed with canagliflozin and not the other SGLT2 inhibitors.^32^ Our result for the composite thrombosis outcome may have been driven by either venous or arterial thrombotic events.

Beyond the outcomes assessed in our analysis, studies have also demonstrated that SGLT2 inhibitors are associated with a reduction in the risk of acute kidney injury as well as renal function decline and progression to end‐stage kidney disease, indicating cardiorenal benefits.^18^, ^33^ Further RCTs focused on other subpopulations, such as those with myocardial infarction, AF and amyloidosis, are currently underway and will likely provide further insights^34^, ^35^, ^36^ but overall, our findings demonstrate the numerous cardiovascular benefits of SGLT2 inhibitors in patients with diabetes, both with and without established cardiovascular disease. Evaluation of these outcomes in non‐diabetic patients will be possible in the coming years as more patients initiate SGLT2 inhibitors for non‐diabetic indications.

In addition to the limitations associated with the retrospective nature of this study, there are other noteworthy points. First, as the TriNetX database comprises information from EMR, data quality is dependent on this and how well these records are updated.

### 4.1 Limitations

Certain conditions may have been underreported, which may have influenced results. Second, data captured within healthcare organizations that are not registered with the TriNetX network will not have been included which may underestimate certain outcomes. Arrhythmias that may have gone unnoticed or cardiac arrests occurring in the community would also contribute to this. Third, as most of the data are based on the United States population, the results may not be directly applicable to the wider population. Lastly, our study does not provide long‐term follow‐up data or a breakdown according to SGLT2 inhibitor drug subtypes.

In conclusion, our findings suggest that SGLT2 inhibitor use was associated with a significant reduction in the risk of incident heart failure and adverse cardiovascular outcomes such as incident AF, cardiac arrest, ischaemic stroke/TIA and arterial and venous thrombosis, although no differences were observed for ventricular arrhythmias.

## CONFLICT OF INTEREST

Ameenathul Mazaya Fawzy: None declared. Jose Miguel Rivera‐Caravaca: Consultancy fees from Idorsia Pharmaceuticals Ltd. Paula Underhill: Employee of TriNetX. Laurent Fauchier: Consultant and speaker activities for AstraZeneca, Bayer, BMS/Pfizer, Boehringer Ingelheim, Medtronic, Novartis, Novo, XO and Zoll. Gregory Y. H. Lip: Consultant and speaker for BMS/Pfizer, Medtronic, Boehringer Ingelheim and Daiichi‐Sankyo. No fees are directly received personally.

### PEER REVIEW

The peer review history for this article is available at https://publons.com/publon/10.1111/dom.14854.

## Supporting information


**APPENDIX S1** Supporting Information

## Data Availability

To gain access to the data in the TriNetX research network, a request can be made to TriNetX (https://live.trinetx.com), but costs may be incurred, a data sharing agreement would be necessary, and no patient identifiable information can be obtained
