# Supplementary material for: Incident heart failure, arrhythmias and cardiovascular outcomes with sodium‐glucose cotransporter 2 (SGLT2) inhibitor use in patients with diabetes: Insights from a global federated electronic medical record database
Source: Diabetes Obes Metab. 2022 Sep 27;25(2):602–10. doi: 10.1111/dom.14854 (PMC10087187; doi:10.1111/dom.14854)
Supplement: Supplementary file 1 — APPENDIX S1 Supporting Information [file DOM-25-602-s001.docx]

**SUPPLEMENTAL DATA**

**Incident heart failure, arrhythmias and cardiovascular outcomes with sodium glucose co-transporter 2 (SGLT2) inhibitor use in diabetic patients:**

**Insights from a global federated electronic medical record database.**

A.M. Fawzy^1^*, J.M. Rivera-Caravaca^1,2^*, Paula Underhill^3^, L. Fauchier^4^**, G.Y.H. Lip^1,5^**

[*joint first authors, **joint senior authors]

^1^Liverpool Centre for Cardiovascular Science, University of Liverpool and Liverpool Heart & Chest Hospital, Liverpool, United Kingdom

^2^Department of Cardiology, Hospital Clínico Universitario Virgen de la Arrixaca, University of Murcia, Instituto Murciano de Investigación Biosanitaria (IMIB-Arrixaca), CIBERCV, Murcia, Spain

^3^TriNetX LLC, London, United Kingdom

^4^Service de Cardiologie, Centre Hospitalier Universitaire Trousseau, Tours, France

^5^Department of Clinical Medicine, Aalborg University, Aalborg, Denmark

**Word count:** 3401 (excluding tables and references)

**Tables/ Figures:** 3/2

**Disclosures:** AMF – None declared. JMR-C –Consultancy fees from Idorsia Pharmaceuticals Ltd**.** PH – Employee of TriNetX. LF –Consultant and speaker activities for AstraZeneca, Bayer, BMS/Pfizer, Boehringer Ingelheim, Medtronic, Novartis, Novo, XO and Zoll. GYHL –Consultant and speaker for BMS/Pfizer, Medtronic, Boehringer Ingelheim, and Daiichi-Sankyo. No fees are directly received personally.

**Corresponding author:**

Prof. Gregory YH Lip.

Liverpool Centre for Cardiovascular Science, Institute of Life Course & Medical Sciences, William Henry Duncan Building, 6 West Derby Street, Liverpool L7 8TX

E-mail: [gregory.lip@liverpool.ac.uk](mailto:gregory.lip@liverpool.ac.uk)

**CONTENTS**

**Supplemental Table 1** – STROBE Statement—checklist of items that should be included in reports of observational studies.

**Supplemental Table 1**. STROBE Statement—checklist of items that should be included in reports of observational studies

|  | Item No. | Recommendation | Page  No. | Relevant text from manuscript |
| --- | --- | --- | --- | --- |
| **Title and abstract** | 1 | (*a*) Indicate the study’s design with a commonly used term in the title or the abstract | 1 | Title on first page |
|  |  | (*b*) Provide in the abstract an informative and balanced summary of what was done and what was found | 2 | Structured abstract |
| Introduction | | | |  |
| Background/rationale | 2 | Explain the scientific background and rationale for the investigation being reported | 4-5 | Introduction section |
| Objectives | 3 | State specific objectives, including any prespecified hypotheses | 4-5 | Introduction section |
| Methods | | | |  |
| Study design | 4 | Present key elements of study design early in the paper | 6 | Methods section under subheading study design and population |
| Setting | 5 | Describe the setting, locations, and relevant dates, including periods of recruitment, exposure, follow-up, and data collection | 6 | Methods section under subheadings study design and population |
| Participants | 6 | (*a*) *Cohort study*—Give the eligibility criteria, and the sources and methods of selection of participants. Describe methods of follow-up  *Case-control study*—Give the eligibility criteria, and the sources and methods of case ascertainment and control selection. Give the rationale for the choice of cases and controls  *Cross-sectional study*—Give the eligibility criteria, and the sources and methods of selection of participants | 6-7 | Methods section under subheadings ‘Study design and population’ and ‘Follow-up and outcomes’. |
|  |  | (*b*)*Cohort study*—For matched studies, give matching criteria and number of exposed and unexposed  *Case-control study*—For matched studies, give matching criteria and the number of controls per case | 6-7 | Methods section under subheadings ‘Study design and population’ and ‘Follow-up and outcomes’. |
| Variables | 7 | Clearly define all outcomes, exposures, predictors, potential confounders, and effect modifiers. Give diagnostic criteria, if applicable | 6-7 | Methods section under subheadings study design and population and ‘Follow-up and outcomes’. |
| Data sources/ measurement | 8* | For each variable of interest, give sources of data and details of methods of assessment (measurement). Describe comparability of assessment methods if there is more than one group | 6-8 | Methods section under subheadings ‘Study design and population’ and ‘Statistical analysis’. |
| Bias | 9 | Describe any efforts to address potential sources of bias | 7-8 | Methods section under subheading ‘Statistical analysis’. |
| Study size | 10 | Explain how the study size was arrived at | 7-8 | Methods section under subheading ‘Statistical analysis’. |

Continued on next page

| Quantitative variables | 11 | Explain how quantitative variables were handled in the analyses. If applicable, describe which groupings were chosen and why | 7-8 | Methods section under subheading ‘Statistical analysis’. |
| --- | --- | --- | --- | --- |
| Statistical methods | 12 | (*a*) Describe all statistical methods, including those used to control for confounding | 7-8 | Methods section under subheading ‘Statistical analysis’. |
|  |  | (*b*) Describe any methods used to examine subgroups and interactions | N/A | N/A |
|  |  | (*c*) Explain how missing data were addressed | 7-8 | Methods section under subheading ‘Statistical analysis’. |
|  |  | (*d*) *Cohort study*—If applicable, explain how loss to follow-up was addressed  *Case-control study*—If applicable, explain how matching of cases and controls was addressed  *Cross-sectional study*—If applicable, describe analytical methods taking account of sampling strategy | N/A | No loss to follow-up |
|  |  | (*e*) Describe any sensitivity analyses | N/A | Not performed. |
| Results | | | | |
| Participants | 13* | (a) Report numbers of individuals at each stage of study—eg numbers potentially eligible, examined for eligibility, confirmed eligible, included in the study, completing follow-up, and analysed | 9 | Results section – 1^st^ and 2^nd^ paragraphs |
|  |  | (b) Give reasons for non-participation at each stage | N/A | N/A |
|  |  | (c) Consider use of a flow diagram | 32 | Figure 1 |
| Descriptive data | 14* | (a) Give characteristics of study participants (eg demographic, clinical, social) and information on exposures and potential confounders | 9 | Results section – 1^st^ and 2^nd^ paragraphs |
|  |  | (b) Indicate number of participants with missing data for each variable of interest | N/A | N/A |
|  |  | (c) *Cohort study*—Summarise follow-up time (eg, average and total amount) | 10 | Results section – total follow-up time indicated |
| Outcome data | 15* | *Cohort study*—Report numbers of outcome events or summary measures over time | 9-12 | Results section |
|  |  | *Case-control study—*Report numbers in each exposure category, or summary measures of exposure | N/A | N/A |
|  |  | *Cross-sectional study—*Report numbers of outcome events or summary measures | N/A | N/A |
| Main results | 16 | (*a*) Give unadjusted estimates and, if applicable, confounder-adjusted estimates and their precision (eg, 95% confidence interval). Make clear which confounders were adjusted for and why they were included | 9-12 | Results section |
|  |  | (*b*) Report category boundaries when continuous variables were categorized | N/A | N/A |
|  |  | (*c*) If relevant, consider translating estimates of relative risk into absolute risk for a meaningful time period | N/A | N/A |

Continued on next page

| Other analyses | 17 | Report other analyses done—eg analyses of subgroups and interactions, and sensitivity analyses | N/A | N/A |
| --- | --- | --- | --- | --- |
| Discussion | | | | |
| Key results | 18 | Summarise key results with reference to study objectives | 12-16 | Discussion section (main body) |
| Limitations | 19 | Discuss limitations of the study, taking into account sources of potential bias or imprecision. Discuss both direction and magnitude of any potential bias | 16 | Discussion section under subheading ‘Limitations’ |
| Interpretation | 20 | Give a cautious overall interpretation of results considering objectives, limitations, multiplicity of analyses, results from similar studies, and other relevant evidence | 12-16 | Discussion section (main) |
| Generalisability | 21 | Discuss the generalisability (external validity) of the study results | 16 | Discussion section – last paragraph |
| Other information | |  | | |
| Funding | 22 | Give the source of funding and the role of the funders for the present study and, if applicable, for the original study on which the present article is based | 18 | After the manuscript text under subheading ‘funding’ |

*Give information separately for cases and controls in case-control studies and, if applicable, for exposed and unexposed groups in cohort and cross-sectional studies.
